# Supplementary material for: Laccase Mediator Cocktail System as a Sustainable Skin Whitening Agent for Deep Eumelanin Decolorization
Source: Int J Mol Sci. 2022 Jun 2;23(11):6238. doi: 10.3390/ijms23116238 (PMC9181290; doi:10.3390/ijms23116238)
Supplement: Supplementary file 1 [file ijms-23-06238-s001.zip › ijms-1744270-supplementary.pdf]

Supplementary Materials

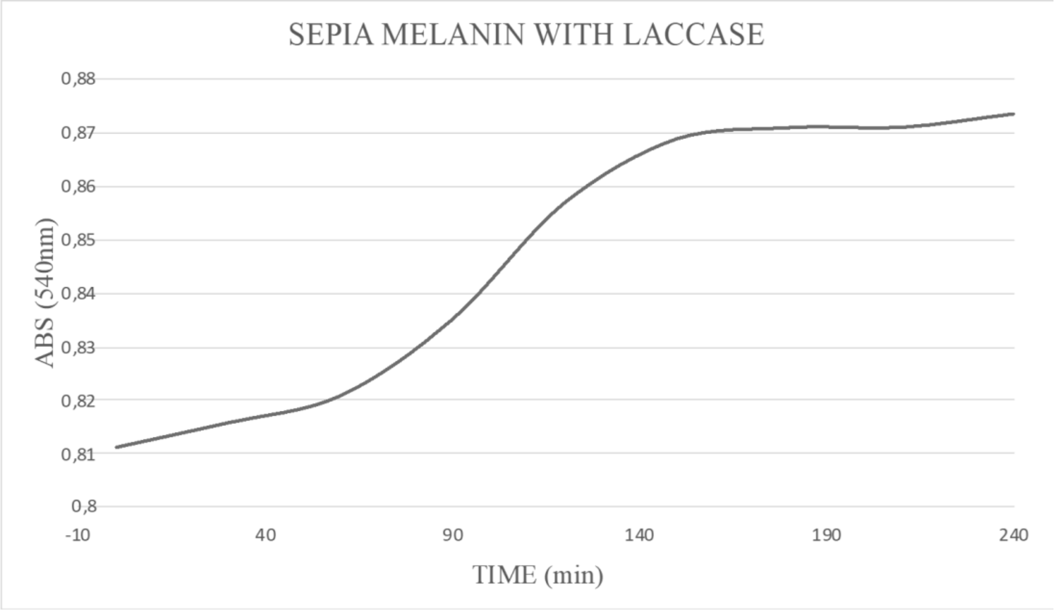

Figure S1. Treatment of *Sepia Melanin* with Laccase from *Trametes versicolor* (0.79 U/mg).
